# Accurate Bracket Placement with an Indirect Bonding Method Using Digitally Designed Transfer Models Printed in Different Orientations—An In Vitro Study

**DOI:** 10.3390/jcm10092002

**Published:** 2021-05-07

**Authors:** Julia Süpple, Julius von Glasenapp, Eva Hofmann, Paul-Georg Jost-Brinkmann, Petra Julia Koch

**Affiliations:** Department of Orthodontics, Dentofacial Orthopedics and Pedodontics, Charité Center for Oral Health Sciences CC3, Charité-Universitätsmedizin Berlin, Corporate Member of Freie Universität Berlin and Humboldt-Universität zu Berlin, Aßmannshauser Straße 4-6, 14197 Berlin, Germany; julia.suepple@charite.de (J.S.); julius.von-glasenapp@charite.de (J.v.G.); eva.hofmann@charite.de (E.H.); paul-g.jost-brinkmann@charite.de (P.-G.J.-B.)

**Keywords:** indirect bonding, transfer accuracy, transfer tray, transfer model, printing orientation, CAD/CAM, digital light processing, 3D printing

## Abstract

Objective: A digital workflow opens up new possibilities for the indirect bonding (IDB) of brackets. We tested if the printing orientation for bracket transfer models on the build platform of a 3D printer influences the accuracy of the following IDB method. We also evaluated the clinical acceptability of the IDB method combining digitally planned and printed transfer models with the conventional fabrication of pressure-molded transfer trays. Materials and Methods: In total, 27 digitally planned bracket transfer models were printed with both 15° and 75° angulation from horizontal plane on the build platform of a digital light processing (DLP) printer. Brackets were temporarily bonded to the transfer models and pressure-molded trays were produced on them. IDB was then performed using the trays on the respective plaster models. The plaster models were scanned with an optical scanner. Digitally planned pre-bonding and scanned post-bonding bracket positions were superimposed with a software and resulted in three linear and three angular deviations per bracket. Results: No statistically significant differences of the transfer accuracy of printed transfer models angulated 15° or 75° on the 3D printer build platform were found. About 97% of the linear and 82% of the angular deviations were within the clinically acceptable range of ±0.2 mm and ±1°, respectively. The highest inaccuracies in the linear dimension occurred in the vertical towards the gingival direction and in the angular dimension in palatal crown torque. Conclusion: For the IDB method used, the printing orientation on the build platform did not have a significant impact on the transfer accuracy.

## 1. Introduction

In the beginning of the 1970s L. F. Andrews introduced the straight-wire appliance and, ever since, accurate bracket placement has been an important objective for orthodontists. Tooth movement was no longer achieved by time-consuming wire-bending, but integrated into the bracket design with a predetermined slot angulation. Accurate bracket positioning of the straight-wire appliance is supposed to result in a correct slot angulation causing the intended tooth movement and treatment outcome [1,2].

Direct bonding is the most frequently used method to attach a straight-wire appliance to the patients’ teeth. Every bracket is bonded separately [3]. To accelerate and facilitate this process and to increase the comfort for patient and orthodontist, Silverman et al. developed indirect bonding (IDB) in 1972. A laboratory-made transfer tray containing the brackets allowed to simultaneously bond them to a group of teeth.

In the conventional IDB workflow, a dental impression is taken to create a dental cast. Brackets are temporarily attached to the model and a transfer tray is produced on top of it [4]. Many different designs and materials for conventional IDB trays have been tested since and show clinical applicability. Most commonly used in the conventional workflow are polyvinyl siloxane and single- or double-vacuum-formed trays, as well as combinations of both [5,6,7,8]. However, these procedures require extra time for taking the impression and extra laboratory steps for producing the tray, which increases the expenses [9]. Thus, only about 12% of the clinicians are using it so far [10].

In recent years, CAD/CAM allowed a digital workflow for IDB. Intraoral scanners provide 3D data of the dental arches that can be imported into software programs. An orthodontic treatment can then be planned virtually, including the precise digital placement of brackets [11]. Based on these data, transfer models or trays can be 3D printed with various printer types for indirect bonding.

The 3D printing of IDB trays was investigated in only a few in vitro studies. The testing of different materials and designs endorses their clinical usability [12,13,14,15,16].

However, the printing of dental models was the objective of various investigations. Especially the digital light processing (DLP) printers, as used in our study, show high precision in printing dental models and are commonly used in orthodontics [17,18].

A frequently mentioned problem in 3D printing that might affect the accuracy is the printing orientation on the build platform. To place as many models as possible, they are often arranged vertically. The staircase effect that is caused by printing in layers appears different depending on the orientation on the platform and affects the surface quality [19]. More knowledge is needed about the impact of this printing parameter on the accuracy. There is limited information available about the testing of different orientations and not for all printer types, materials, and object geometries. This has led to inconsistent recommendations [19,20,21].

**Figure 1:**
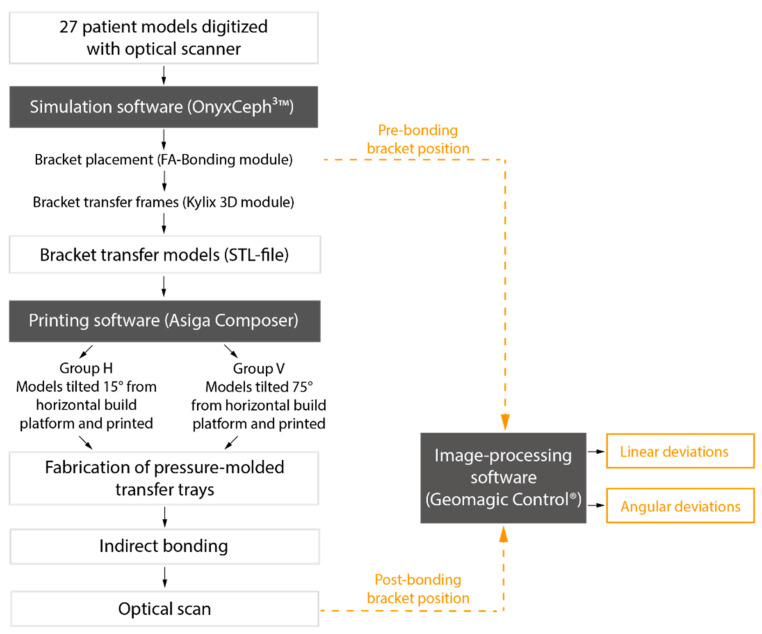
Flow chart of the IDB workflow and analysis of transfer accuracy with bracket position deviations.

For our study, we 3D printed transfer models with frames for every bracket position to produce IDB trays. Our aim was to test different printing orientations on the build platform in two groups. We asked whether the printing orientation influences the accuracy and if the IDB method used—combining both the conventional and digital workflow—transfers the brackets with clinically acceptable accuracy (Figure 1).

## 2. Materials and Methods

**Figure 2:**
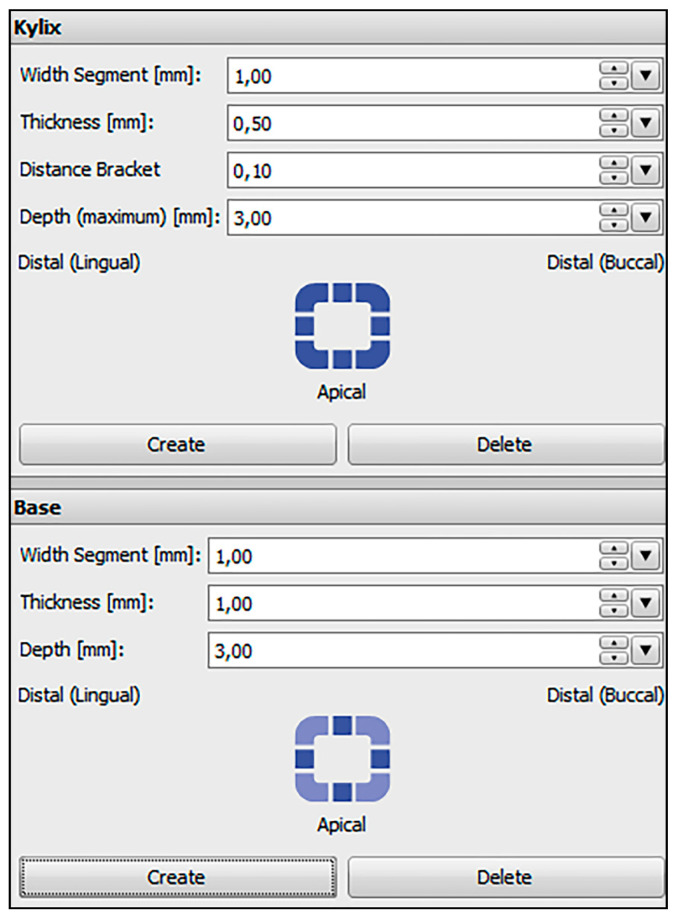
Parameters used in the OnyxCeph^3^™ Kylix 3D module (Image Instruments, Chemnitz, Germany).

Plaster models of 27 patients with permanent dentition and in need of orthodontic treatment were digitized with an optical scanner (TRIOS^®^3W, 3Shape, Copenhagen, Denmark). The scans were saved as standard tessellation language (STL) files and imported to the treatment simulation software OnyxCeph^3^™ (Image Instruments, Chemnitz, Germany). All bracket positions were determined and virtually placed using the OnyxCeph^3^™ FA-Bonding module. The patient models were planned with metal brackets (0.018-inch slots) for incisors, canines and premolars (discovery^®^ smart, Dentaurum, Ispringen, Germany) and metal tubes for the first and second molars (Ortho-Cast M-Series, Dentaurum, Ispringen, Germany) in the upper and lower jaw. Eight patient models got ceramic brackets (discovery^®^ pearl, Dentaurum, Ispringen, Germany) from the second premolar on one side to the second premolar on the contralateral side in the upper jaw. The pre-bonding bracket positions were set for every tooth including the first and second molars. Based on the determined bracket positions, frames were virtually created around every bracket in the OnyxCeph^3^™ Kylix 3D module (Image Instruments, Chemnitz, Germany). All transfer models including the bracket frames were exported as STL files. The parameters used for the dimension of the frames are shown in Figure 2.

### 2.1. Printing the Bracket Transfer Models

**Figure 3:**
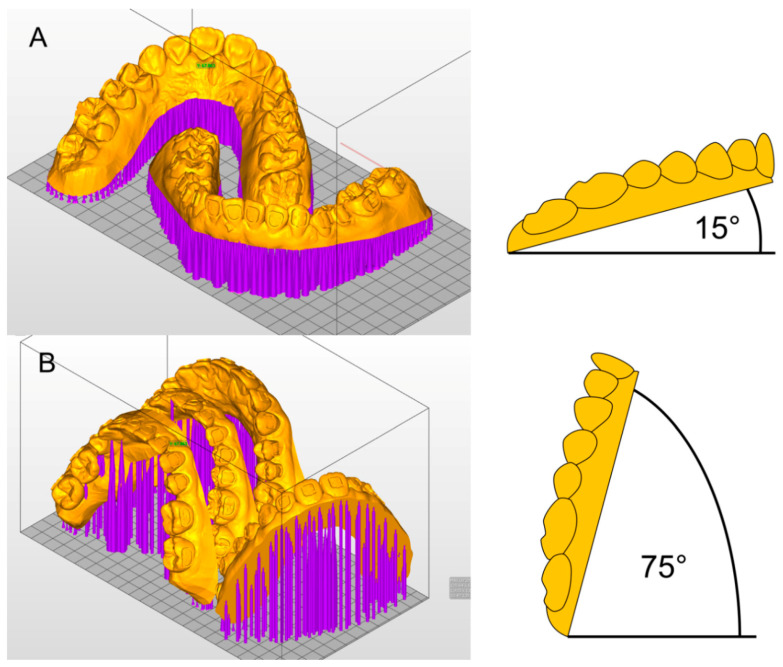
Printing orientation in (**A**) Group H and (**B**) Group V in Asiga MAX™ printer software (Asiga Composer, Scheu Dental, Iserlohn) and model angulations.

The STL files of the transfer models were imported to the Asiga MAX™ printer software (Asiga Composer, Scheu Dental, Iserlohn, Germany). All 27 patient models were sent to a 3D printer with DLP technology (Asiga MAX™, Scheu Dental, Iserlohn, Germany). They were oriented horizontally or vertically on the build platform of the printer (Figure 3).

(A) Group H: 27 models were tilted 15° from the horizontal build platform and printed. One patient model per print (upper and lower jaw) was placed on the build platform and printed in 30 to 45 min.

(B) Group V: another 27 models were tilted 15° from the vertical line (75° from the horizontal build platform), which allowed the placement of two sets of patient models on the platform. One print took 75 to 90 min.

**Figure 4:**
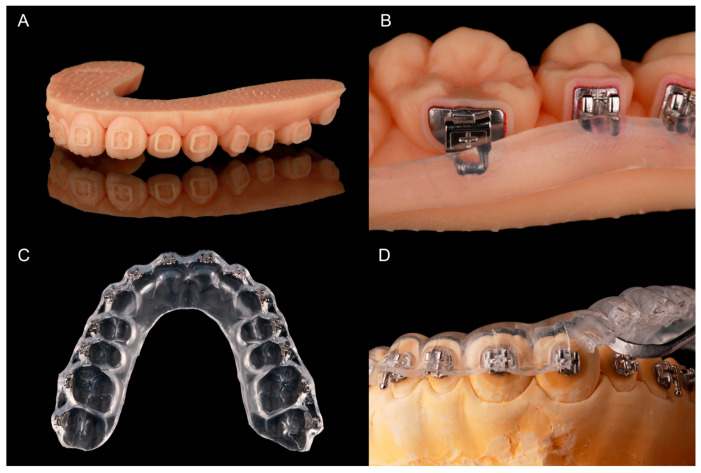
Fabricating the pressure-molded transfer trays: (**A**) printed model with frames for brackets and tubes; (**B**) provisory bonded brackets and tubes with silicone for blocking-out; (**C**) pressure-molded transfer tray with embedded brackets and tubes; (**D**) tray removal with a scaler after bonding.

Light-curing methacrylate-based resin (IMPRIMO^®^ LC model, Scheu Dental, Iserlohn, Germany) was used for printing. A slice thickness of 0.05 mm was chosen. Support structures were added automatically and without connection to the frames. To attach the support structures securely to the build platform, a 0.3 mm thick base plate was created. After printing, the models were detached from the build platform and the support structures were removed with a scraper. As recommended by the printer producer, the models were then immersed into an ultrasonic cleaning device (IMPRIMO^®^ Clean, Scheu Dental, Iserlohn, Germany) filled with a butyldiglycol-based detergent solution (IMPRIMO^®^ Cleaning Liquid, Scheu Dental, Iserlohn, Germany) for 10 min. The models were then light-cured for five minutes using a resin-specific program with a wavelength of 405 nm in a nitrogen environment (IMPRIMO^®^ Cure, Scheu Dental, Iserlohn, Germany). A finished transfer model is shown in Figure 4A.

### 2.2. Fabricating the Pressure-Molded Transfer Trays

All brackets were temporarily bonded into their frames with a water-soluble adhesive (Ortho Laboratory Adhesive for Indirect Bonding, 3M™ Unitek, St. Paul, MN, USA). The brackets were then blocked out up to the middle of the slots with a silicone (SIL-KITT^®^, Scheu Dental, Iserlohn, Germany). The hooks of the molar tubes were also covered (Figure 4B). The models were placed into a pressure molding machine (BIOSTAR^®^, Scheu Dental, Iserlohn, Germany) to produce an ethylene-vinyl acetate tray (BIOPLAST^®^ 2.0 × 125 mm, Scheu, Iserlohn, Germany). The tray—containing the brackets—was cut into shape (Figure 4C) and put into water for 30 min to dissolve the adhesive. To allow an easy removal of the tray after IDB, it was cut with a scalpel from the margin to the middle of the brackets or tubes. 

### 2.3. Bracket Bonding

Plaster models for every patient were cast using silicone forms of the initial patient situation.

The facial tooth surfaces of the plaster models were cleaned with isopropanol and Transbond™ XT Primer (3M Unitek Deutschland, Neuss, Germany) was applied on the expected bracket positions. The bracket bases were cleaned with a cotton pellet soaked in acetone and Transbond™ XT (3M Unitek Deutschland, Neuss, Germany) was allocated to them. Afterwards, the tray was put on the model and material excess of the composite was removed with a dental probe. Every bracket was light-cured with 3200 mW/cm^2^ in the extra power light polymerization mode (Valo^®^ Cordless, Ultradent Products, Cologne, Germany) for 12 s while holding the tray in place with slight and even occlusal pressure. The tray was then removed with the help of a scaler (Figure 4D).

### 2.4. Comparing Pre- and Post-Bonding Bracket Position

**Figure 5:**
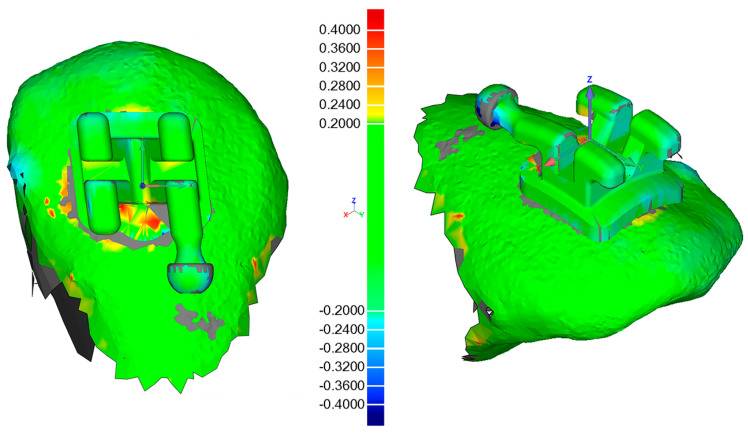
Illustration of the output from the superimposition in Geomagic Control^®^ software (3D Systems Inc., Rock Hill, SC, USA) for tooth 13 of a random patient.

A scanning powder (METAL-POWDER Dry blue, R-dental Dentalerzeugnisse, Hamburg, Germany) was sprayed on the plaster models to avoid reflections from the metal surfaces. Every model was scanned to digitize the post-bonding bracket positions (TRIOS^®^3W, 3Shape, Copenhagen, Denmark). Both pre- and post-bonding STL data were imported to Geomagic Control^®^ (3D Systems Inc., Rock Hill, SC, USA). Every tooth was cut out and saved both in pre- and post-bonding situation. In the image-processing software, the corresponding teeth were superimposed with a local best-fit alignment (Figure 5) and resulted in three linear and three angular measurements for each bracket.

### 2.5. Statistical Analysis

All measurements were inserted into the SPSS software (IBM SPSS Statistics 27, Armonk, NY, USA). Means and standard deviations of the absolute numbers were calculated for the tooth groups (incisors/canines/premolars/molars) in Groups H and V.

A linear mixed model was conducted two times: one using all linear dimensions (mesiodistal/vertical/orovestibular) as dependent variable and one using all angular dimensions (torque/rotation/tip). The Groups H and V, upper and lower jaw, as well as the tooth groups (incisor/canine/premolar/molar) were set as factors.

## 3. Results

We analyzed the transfer accuracy of 1453 brackets and tubes, 729 in Group H (15° angulation) and 724 in Group V (75° angulation). Overall, 11 teeth of the 27 patient models were missing due to agenesis or early tooth loss. In total, 15 brackets in Group H were lost during the transfer procedure and 17 in Group V. One bracket position analysis in Group H and four in Group V were considered invalid due to a failing superimposition in the Geomagic software.

The linear mixed model shows no significant difference between Groups H and V in the linear or angular dimensions (Table 1).

However, deviations in the tooth groups (of both Group H and V) are significant for every dimension: In the linear dimension the molars show the worst and the canines the best results of transfer accuracy, while in the angular dimension it is the other way round.

A significant difference between upper and lower jaw exists in the overall linear dimension, showing better transfer accuracy in the lower jaw.

Table 2 presents the means and standard deviations of the transfer accuracy in all dimensions as calculated with absolute numbers. The best linear transfer accuracy is achieved in the orovestibular direction with a mean deviation of 0.03 mm in Group H and 0.02 mm in Group V. The vertical dimension shows a mean deviation of 0.08 mm in Groups H and V and is, therefore, the most inaccurate. The overall deviations for each of the three linear directions are statistically significant (Table 1).

No significant difference was found for the angular dimensions (Table 1). A mean deviation of 0.55° in Group H and 0.56° in Group V reveals that tip is transferred most accurately. A mean of 0.65° in Group H and 0.67° in Group V identifies torque to be the most inaccurately transferred angular dimension (Table 2).

We considered linear deviations of ±0.2 mm and angular deviations of ±1° clinically acceptable. The percentage of transfers outside of the acceptable range is presented in Table 3.

The greatest deviations in the linear dimension were found in the vertical direction. In Group H 3.7% and in Group V 5.4% of the brackets were transferred more than 0.2 mm too far gingival. All vestibular deviations were within the acceptable range. Therefore, the most accurate linear dimension is orovestibular (Table 3).

The lowest as well as the highest percentage of transfer failures in the angular dimension is shown in torque. In Group H 18.9% and in Group V 18.1% were transferred with a clinically unacceptable palatal crown torque, while only 1.8% in Group H and V were transferred with too much labial crown torque (Table 3).

## 4. Discussion

The aim of our in vitro study was to test two different printing orientations on the build platform in a digital IDB workflow. We also investigated the transfer accuracy within the clinical requirements for the IDB method.

We found no statistically significant difference in the transfer accuracy of IDB trays based on transfer models which were 3D printed with a 15° (Group H) and 75° (Group V) angulation from the horizontal build platform. However, significant differences were found when comparing all tooth groups regardless of Group H or V: Incisors showed a high transfer accuracy, whilst the accuracy of the different directions was more inconsistent for canines, premolars, and molars. When comparing the transfer accuracy within the jaws, the lower jaw generally displayed better results. In general, the highest inaccuracies were found in the vertical direction and for torque.

To evaluate the usability of our method we had to define a range for clinical acceptability. The American Board of Orthodontics has suggested a maximum deviation of 0.5 mm and 2° for bracket positioning [22]. As previously explained by Schmid et al., these limits need to consider bracket deviations in opposite directions of neighboring teeth [5]. Therefore, we defined this range for our analysis: a maximum deviation of ±0.2 mm linear and ±1° angular.

The linear transfer accuracy was within that clinically acceptable range in 97% of the cases in the mesiodistal, 95% in the vertical and 99.7% in the orovestibular direction. The angular dimension was within the range in 79.7% of the cases for torque, 83.2% for rotation and 82.7% for tip.

**Figure 6:**
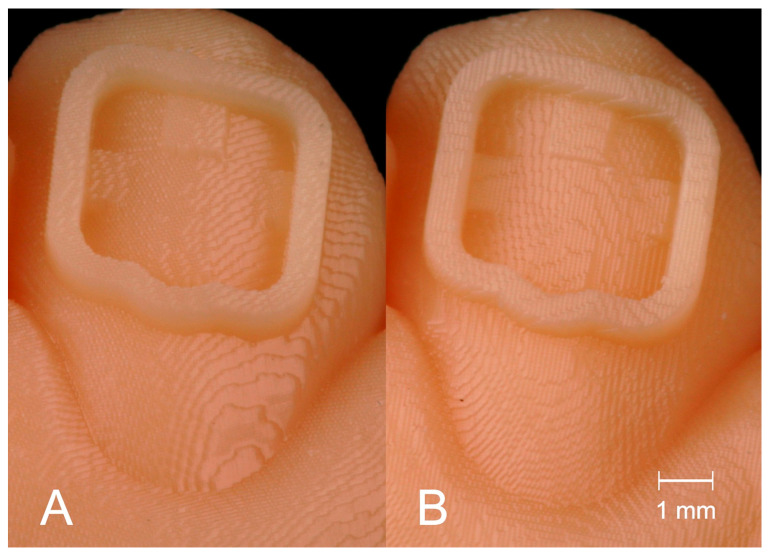
Staircase effect on the surface of the transfer models in Group H (**A**) and Group V (**B**).

The positioning of dental models on the build platform of a printer is often mentioned to influence the precision. The staircase effect that occurs on the surface of a printed object has a great impact on accuracy and appears differently depending on the printing direction [19]. Figure 6 shows the staircase effect on the transfer models with bracket frames in Groups H (A) and V (B). Previous studies found different ideal printing directions. Hada et al. compared SLA printed dentures in three different angulations (0°/45°/90°) on the build platform. Unkovskiy et al. used the same printer type and angulations to produce specimens. An angulation of 45° achieved the best results in both studies [19,23]. Shim et al. printed specimens of different angulations and identified 90° to be the best orientation for precise manufacturing [20].

We used a DLP printer and a slice thickness of 0.05 mm instead of the more commonly used 0.1 mm. This may explain why the accuracy of our printed transfer models with 15° or 75° angulation did not differ significantly. The DLP printer used, has a xy-resolution of 0.062 mm. Together with a slice thickness (=z-resolution) of 0.05 mm, a resolution consisting of nearly cubic elements—similar to a voxel—is created. Therefore, the same outcome should occur, no matter what position the object is printed in.

Nevertheless, different support structures are required for different angulations in order to avoid detachment from the build platform during printing. In addition, the IDB tray fabrication on the transfer models may have hidden differences between our test groups. Testing the accuracy of the transfer model itself would be needed to reveal a difference between the 3D prints.

In the study by Arnold et al. the arrangement of objects on the build platform of SLA printers was found to have an impact on accuracy. They discovered that in the front of the platform the most accurate models are produced [21]. In contrast to this, Unkovskiy et al. found that objects placed in the center of the build platform are more accurate than those placed at the border of it [23]. In our study, we focused on arranging our transfer models according to the model size and limited space on the build platform. We did not focus on the arrangement on it. Further investigations about how the placement on the platform areas influences accuracy are needed.

We used a specific DLP printer and followed the working steps that were recommended by the manufacturer. Hazeveld et al. analyzed the accuracy of printed dental models. They concluded that DLP printers were appropriate for orthodontic requirements and show a high accuracy when compared with two other types of 3D printed and conventional plaster models [24]. Yet, other printer types and manufacturers could be tested with the transfer models of the OnyxCeph^3^™ Kylix 3D module (Image Instruments GmbH, Chemnitz, Germany) to further evaluate this workflow.

A single pressure-molded tray provides an easy and fast laboratory workflow. We chose this type of transfer tray to evaluate a work routine that orthodontists would realistically want to use. However, we had to deal with the fact that pressure-formed IDB trays showed a worse transfer accuracy than other types of trays in previous studies. Dörfer et al., Castilla et al., as well as Schmid et al., have reported a worse transfer with single vacuum-formed trays than with polyvinylsiloxane or double layer IDB trays [5,6,8]. Therefore, our results for the transfer accuracy might have been better with other trays. We focused on single pressure-molded trays in this investigation, but the same transfer models could be tested with various other tray materials in future studies.

In our, and in previous studies, the greatest transfer inaccuracies in the linear dimension were found in the vertical direction [5,6,7,8,16]. Inconsistent pressure on the tray during the bonding process is often mentioned as an explanation for this [5,6,7,16]. Most authors found that the deviation was towards the occlusal direction. However, Grünheid et al. found gingival transfer errors to be most common in the vertical direction and explained this with too much finger pressure on the transfer tray during bonding [7]. The same mechanism seems to apply to our results, since the biggest vertical error in our study occurred in the gingival direction.

Dörfer and coworkers observed a thermoplastic shrinkage when using pressure-formed transfer trays, resulting in transfer inaccuracy (especially in the mesiodistal direction) and increasing in the posterior direction [8]. The effects of thermoplastic shrinkage may have influenced our results as well.

The high transfer accuracy in the orovestibular direction might be explained by the frames for the bracket positions that were created in the OnyxCeph^3^™ Kylix 3D module (Image Instruments GmbH, Chemnitz, Germany). The frames of the printed transfer models appear as negative spaces around the brackets in the tray. Any excess of bonding material can, therefore, flow into these spaces. This way, the individual bracket base can get the right thickness during bonding. However, it is hard to remove the excess completely before light-curing and makes removal of cured material necessary.

For the angular dimensions torque, rotation and tip, Niu et al. found that they were generally less accurate than the linear dimensions [16]. This supports our results. Torque showed the worst transfer accuracies of all angular dimensions, and the same result was found in previous studies [5,7,14,16]. Nui et al. refer to an excess of bonding material or the transfer tray design to explain the outcome for torque [16]. Most investigations though, lack an explanation for these results.

We also assume that the tray design plays an important role—especially regarding the bracket attachment in the tray. Since the brackets were completely surrounded by the frames in the transfer model, they were only held in the transfer tray with the bracket wings. Therefore, a great freedom in the angular dimensions appears in our IDB method. That might explain why the angular transfer accuracy was worse than the linear and did not significantly differ between torque, rotation, and tip.

Significant differences between the tooth groups and jaws were found in our study, as well as in others testing IDB workflows [5,16]. The shape of the tooth seems to play an important role for the transfer accuracy, as well as the accessibility that is worse in the posterior direction [6,7,25,26]. Castilla et al. explained that the differences in thickness of a vacuum-formed transfer tray result in different accuracy outcomes in the dental arch. As a reason for this, they mention the difference in crown length of incisors and molars, respectively [6]. A plane facial surface and good clinical accessibility should lead to high bracket transfer accuracy. The generally good results we found for the incisors confirm this hypothesis.

We evaluated the accuracy of 1453 brackets and tubes placed with IDB, while other studies analyzed between 136 and 300 brackets [5,6,7,8,14,15,16]. Most other studies investigated IDB from the central incisor to the first molar. Some were even skipping the molars completely and using a transfer tray including incisors, canines, and premolars only. Our transfer trays included the second molars, making an assumption for accuracy in the posterior direction possible. In addition, we chose 27 patient models with different malocclusions. Various clinical challenges for IDB, such as crowding, rotation of teeth or spaces are included in our analysis.

Nevertheless, an in vitro study lacks some conditions that would occur in vivo: There was no soft tissue, so the tray and brackets could not displace gingival tissue in order to reach the right bracket placement. Common clinical challenges such as saliva, muscle movement, restricted mouth opening, or patient compliance were not taken into account. The clinical outcome of accuracy might differ and should be tested in subsequent in vivo studies.

The study analyzed both the transfer accuracy of the IDB method and the influence of different printing orientations of transfer models on the accuracy of a following IDB workflow. Both topics were investigated simultaneously. Therefore, the IDB workflow might have covered inaccuracies of the transfer models of Group H and V.

The software Geomagic Control allowed us to superimpose the pre- and post-bonding bracket positions of the whole bracket surface and the corresponding tooth. This method may increase the accuracy of the analysis compared to other optical or point-based methods used in previous studies [27].

When a slice thickness close to the xy-resolution of the printer is used, the accuracy of models placed with a 15° or 75° angulation on the build platform does not significantly differ. We found no statistically significant differences between the tested Groups H and V.

Accurate bracket placement is possible with a single pressure-molded transfer tray. Other tray materials could be used for our workflow and might lead to even better transfer accuracy.

The printed OnyxCeph^3^™ Kylix 3D module (Image Instruments GmbH, Chemnitz, Germany) transfer models offer a digital workflow that is combined with the advantages of a conventional workflow. It provides a flexible method that can be adapted to the user’s preferences.

## 5. Conclusions

The printing orientation of the transfer models angulated 15° and 75° from the build platform for the fabrication of conventional IDB trays did not significantly influence the transfer accuracy: 97% of the linear and 82% of the angular deviations were within the clinically acceptable range of ±0.2 mm and ±1°.

The most frequent bracket position deviations were found in the vertical towards the gingival direction (for the linear dimensions) and in palatal crown torque (for the angular dimensions).

## Figures and Tables

**Table 1:** Mixed model: Fixed effects for the linear and angular dimension.

	*p*-Value	
Factors	Linear Dimension	Angular Dimension
Groups (H/V)	0.60	0.71
Dimensions (linear/angular)	0.00 *	0.24
Tooth groups (incisors/canines/premolars/molars)	0.01 *	0.00 *
Jaws (upper/lower)	0.00 *	0.06
Groups × dimensions ^a^	0.17	0.34
Groups × tooth groups ^a^	0.06	0.04 *
Groups × upper and lower jaw ^a^	0.88	0.78
Dimensions × tooth groups ^a^	0.27	0.00 *
Dimensions × upper and lower jaw ^a^	0.98	0.71
Tooth groups × upper and lower jaw ^a^	0.32	0.41

* *p* < 0.05 indicates statistical significance. ^a^ interaction between the factors (×).

**Table 2:** Differences between pre- and post-bonding positions in Group H and V for different tooth types.

			Mean ^b^ ± SD					
Tooth Type	Group	n ^a^	Mesiodistal (mm)	Vertical (mm)	Orovestibular (mm)	Torque (°)	Rotation (°)	Tip (°)
Incisors	H	210	0.05 ± 0.04	0.07 ± 0.05	0.02 ± 0.02	0.49 ± 0.36	0.53 ± 0.47	0.77 ± 0.61
	V	209	0.05 ± 0.04	0.07 ± 0.06	0.02 ± 0.02	0.50 ± 0.40	0.55 ± 0.46	0.79 ± 0.60
Canines	H	107	0.07 ± 0.06	0.07 ± 0.07	0.03 ± 0.05	0.64 ± 0.55	0.82 ± 0.80	0.72 ± 0.65
	V	106	0.06 ± 0.06	0.07 ± 0.07	0.03 ± 0.03	0.64 ± 0.53	0.72 ± 0.69	0.67 ± 0.54
Premolars	H	207	0.07 ± 0.07	0.09 ± 0.07	0.02 ± 0.02	0.80 ± 0.59	0.67 ± 0.69	0.55 ± 0.48
	V	206	0.06 ± 0.07	0.08 ± 0.06	0.02 ± 0.05	0.74 ± 0.58	0.59 ± 0.67	0.56 ± 0.54
Molars	H	205	0.06 ± 0.07	0.09 ± 0.06	0.03 ± 0.03	0.68 ± 0.49	0.56 ± 0.62	0.23 ± 0.26
	V	203	0.06 ± 0.07	0.10 ± 0.08	0.03 ± 0.03	0.79 ± 0.68	0.56 ± 0.64	0.26 ± 0.37
Total	H	729	0.06 ± 0.06	0.08 ± 0.06	0.03 ± 0.03	0.65 ± 0.51	0.62 ± 0.64	0.55 ± 0.55
	V	724	0.06 ± 0.06	0.08 ± 0.07	0.02 ± 0.04	0.67 ± 0.57	0.59 ± 0.61	0.56 ± 0.55

^a^ number of brackets used for analysis. ^b^ mean calculated with absolute numbers of transfer deviations.

**Table 3:** Prevalence of bracket transfers outside of the clinically acceptable range in Group H and V for different tooth types.

		Mesiodistal (%)		Vertical (%)		Orovestibular (%)		Torque (%)		Rotation (%)		Tip (%)	
Tooth Type	Group	Mesial	Distal	Occlusal	Gingival	Oral	Vestibular	PCT	LCT	MR	DR	MCT	DCT
Incisors	H	0.5	0.0	0.5	1.4	0.0	0.0	7.1	1.0	5.2	8.1	15.7	17.1
	V	0.0	1.0	0.5	2.9	0.0	0.0	8.6	1.0	6.2	6.7	18.2	12.0
Canines	H	3.7	0.0	0.0	5.6	0.9	0.0	15.0	1.9	5.6	22.4	15.9	9.3
	V	2.8	0.0	0.9	3.8	0.0	0.0	17.0	2.8	6.6	14.2	7.5	15.1
Premolars	H	2.9	1.0	1.0	3.9	0.0	0.0	31.4	2.9	3.4	15.0	6.8	5.8
	V	1.9	0.5	0.5	4.4	0.5	0.0	22.8	2.4	8.3	7.8	9.7	4.9
Molars	H	4.9	1.5	0.0	4.9	0.5	0.0	20.5	1.5	11.2	4.4	0.5	1.5
	V	0.5	3.0	0.0	9.9	0.5	0.0	23.6	1.5	12.8	3.9	2.0	2.5
Total	H	1.9	1.6	0.4	3.7	0.3	0.0	18.9	1.8	6.4	11.1	8.0	9.3
	V	1.1	1.2	0.4	5.4	0.3	0.0	18.1	1.8	8.7	7.3	9.7	7.7

PCT = Palatal crown torque, LCT = Labial crown torque, MR = Mesiorotation, DR = Distorotation, MCT = Mesial crown tip, DCT = Distal crown tip.

## Data Availability

The data underlying this article will be shared on reasonable request to the corresponding author.

## References

[1] Andrews L.F. (1976). The straight-wire appliance, origin, controversy, commentary. J. Clin. Orthod..

[2] Miethke R.R., Melsen B. (1999). Effect of variation in tooth morphology and bracket position on first and third order correction with preadjusted appliances. Am. J. Orthod. Dentofac. Orthop..

[3] Newman G.V. (1965). Epoxy adhesives for orthodontic attachments: Progress report. Am. J. Orthod. Dentofac. Orthop..

[4] Silverman E., Cohen M., Gianelly A.A., Dietz V.S. (1972). A universal direct bonding system for both metal and plastic brackets. Am. J. Orthod..

[5] Schmid J., Brenner D., Recheis W., Hofer-Picout P., Brenner M., Crismani A.G. (2018). Transfer accuracy of two indirect bonding techniques—an in vitro study with 3D scanned models. Eur. J. Orthod..

[6] Castilla A.E., Crowe J.J., Moses J.R., Wang M., Ferracane J.L., Covell D.A. (2014). Measurement and comparison of bracket transfer accuracy of five indirect bonding techniques. Angle Orthod..

[7] Grünheid T., Lee M.S., Larson B.E. (2016). Transfer accuracy of vinyl polysiloxane trays for indirect bonding. Angle Orthod..

[8] Dörfer S., König M., Jost-Brinkmann P. (2006). Übertragungsgenauigkeit beim indirekten Platzieren von Brackets. Kieferorthopädie.

[9] Czolgosz I., Cattaneo P.M., Cornelis A.M. (2020). Computer-aided indirect bonding versus traditional direct bonding of orthodontic brackets: Bonding time, immediate bonding failures, and cost-minimization. A randomized controlled trial. Eur. J. Orthod..

[10] Sheridan J.J. (2004). The Readers’ Corner. 1. Do you use indirect bonding?. J. Clin. Orthod..

[11] De Oliveira N.S., Rossouw E., Lages E.M.B., Macari S., Pretti H. (2019). Influence of clinical experience on accuracy of virtual orthodontic attachment bonding in comparison with the direct procedure. Angle Orthod..

[12] Duarte M.E.A., Gribel B.F., Spitz A., Artese F., Miguel J.A.M. (2020). Reproducibility of Digital Indirect Bonding Technique Using Three-dimensional (3d) Models and 3d-printed Transfer Trays. Angle Orthod..

[13] Xue C., Xu H., Guo Y., Xu L., Dhami Y., Wang H., Liu Z., Ma J., Bai D. (2020). Accurate bracket placement using a comput-er-aided design and computer-aided manufacturing–guided bonding device: An in vivo study. Am. J. Orthod. Dentofac. Orthop..

[14] Pottier T., Brient A., Turpin Y.L., Chauvel B., Meuric V., Sorel O., Brezulier D. (2020). Accuracy evaluation of bracket reposi-tioning by indirect bonding: Hard acrylic CAD/CAM versus soft one-layer silicone trays, an in vitro study. Clin. Oral Investig..

[15] Zhang Y., Yang C., Li Y., Xia D., Shi T., Li C. (2020). Comparison of three-dimensional printing guides and double-layer guide plates in accurate bracket placement. BMC Oral Health.

[16] Niu Y., Zeng Y., Zhang Z., Xu W., Xiao L. (2021). Comparison of the transfer accuracy of two digital indirect bonding trays for labial bracket bonding. Angle Orthod..

[17] Sherman S.L., Kadioglu O., Currier G.F., Kierl J.P., Li J. (2020). Accuracy of digital light processing printing of 3-dimensional dental models. Am. J. Orthod. Dentofac. Orthop..

[18] Kim S.-Y., Shin Y.-S., Jung H.-D., Hwang C.-J., Baik H.-S., Cha J.-Y. (2018). Precision and trueness of dental models manufactured with different 3-dimensional printing techniques. Am. J. Orthod. Dentofac. Orthop..

[19] Hada T., Kanazawa M., Iwaki M., Arakida T., Soeda Y., Katheng A., Otake R., Minakuchi S. (2020). Effect of Printing Direction on the Accuracy of 3D-Printed Dentures Using Stereolithography Technology. Materials.

[20] Shim J.S., Kim J.-E., Jeong S.H., Choi Y.J., Ryu J.J. (2020). Printing accuracy, mechanical properties, surface characteristics, and microbial adhesion of 3D-printed resins with various printing orientations. J. Prosthet. Dent..

[21] Arnold C., Monsees D., Hey J., Schweyen R. (2019). Surface Quality of 3D-Printed Models as a Function of Various Printing Parameters. Materials.

[22] Casko J.S., Vaden J.L., Kokich V.G., Damone J., James R.D., Cangialosi T.J., Riolo M.L., Owens S.E., Bills E.D. (1998). Ob-jective grading system for dental casts and panoramic radiographs. American Board of Orthodontics. Am. J. Orthod. Dentofac. Orthop..

[23] Unkovskiy A., Bui P.H.-B., Schille C., Geis-Gerstorfer J., Huettig F., Spintzyk S. (2018). Objects build orientation, positioning, and curing influence dimensional accuracy and flexural properties of stereolithographically printed resin. Dent. Mater..

[24] Hazeveld A., Slater J.J.H., Ren Y. (2014). Accuracy and reproducibility of dental replica models reconstructed by different rapid prototyping techniques. Am. J. Orthod. Dentofac. Orthop..

[25] Kim J., Chun Y.-S., Kim M. (2018). Accuracy of bracket positions with a CAD/CAM indirect bonding system in posterior teeth with different cusp heights. Am. J. Orthod. Dentofac. Orthop..

[26] Palone M., Spedicato G.A., Lombardo L. (2020). Analysis of tooth anatomy in adults with ideal occlusion: A preliminary study. Am. J. Orthod. Dentofac. Orthop..

[27] Koch P.J. (2020). Measuring the accuracy of a computer-aided design and computer-aided manufacturing–based indirect bonding tray. Am. J. Orthod. Dentofac. Orthop..

